# Varicella Zoster Meningitis in a Young, Immunocompetent Patient Despite Initiation of Antiviral Therapy

**DOI:** 10.7759/cureus.39980

**Published:** 2023-06-05

**Authors:** Yashitha Chirumamilla, Sania Ajmal, Bhawuk Subedi, Ghassan Bachuwa, Basim Towfiq

**Affiliations:** 1 Internal Medicine, Hurley Medical Center, Flint, USA; 2 Internal Medicine/Pediatrics, Hurley Medical Center, Flint, USA

**Keywords:** herpes zoster meningitis, herpes zoster, acyclovir, viral meningitis, varicella zoster reactivation, varicella-zoster virus

## Abstract

Varicella zoster virus (VZV) reactivation, also known as herpes zoster is common in older adults and immunocompromised individuals and often causes a painful, vesicular rash limited to a dermatomal distribution. On occasion, it can lead to various neurological complications as well. Here we present the case of a young, immunocompetent male in his 20's with a history of primary varicella infection who presented with complaints of a painful rash in the S3-S4 dermatomal distribution. Despite being initiated on the standard oral antiviral dose for two days, he developed a headache and neck stiffness. He was diagnosed with VZV meningitis through the lumbar puncture and cerebrospinal fluid polymerase chain reaction (PCR) assay analysis. The patient reported significant improvement in symptoms following intravenous acyclovir and was discharged with additional oral valacyclovir at a higher-than-standard dosage. Our case highlights that even in relatively low-risk patients, physicians must maintain a high level of clinical suspicion for the complications of VZV reactivation even after beginning the oral antiviral medication.

## Introduction

Varicella zoster virus (VZV) is responsible for two clinically distinct disease processes. The first is a primary varicella infection, also known as chickenpox, characterized by vesicular lesions on an erythematous base [1]. The second is VZV reactivation, also known as herpes zoster or shingles which typically causes a painful, vesicular rash limited to a dermatomal distribution but can further lead to neurological complications. After primary infection, the virus remains latent in the cranial nerve dorsal root and autonomic ganglion and often remains dormant for decades or until the host’s immune system is otherwise compromised [2]. Therefore, herpes zoster is commonly a disease encountered in elderly or immunosuppressed patients.

## Case presentation

We present the case of a 26-year-old Caucasian male with a past medical history of attention deficit hyperactivity disorder and confirmed primary varicella infection in childhood and a relevant family history of herpes zoster in his sister during childhood. He presented to the emergency department with complaints of a headache, extreme sensitivity to light, and neck stiffness for the past 12 hours. He also reported a painful rash that began four days earlier which was initially located over the buttocks and later spread to the scrotum and penis. The patient had been in a monogamous relationship with no history of sexually transmitted infections. He was seen at a different facility two days earlier for the painful rash and was suspected of having a herpes zoster infection. He was discharged on oral valacyclovir 1 gram three times daily for seven days. He reported that despite being compliant with the medications, he continued to have worsening pain at the site of the rash and new-onset headaches which precipitated him to seek medical attention once again. 

**Figure 1:**
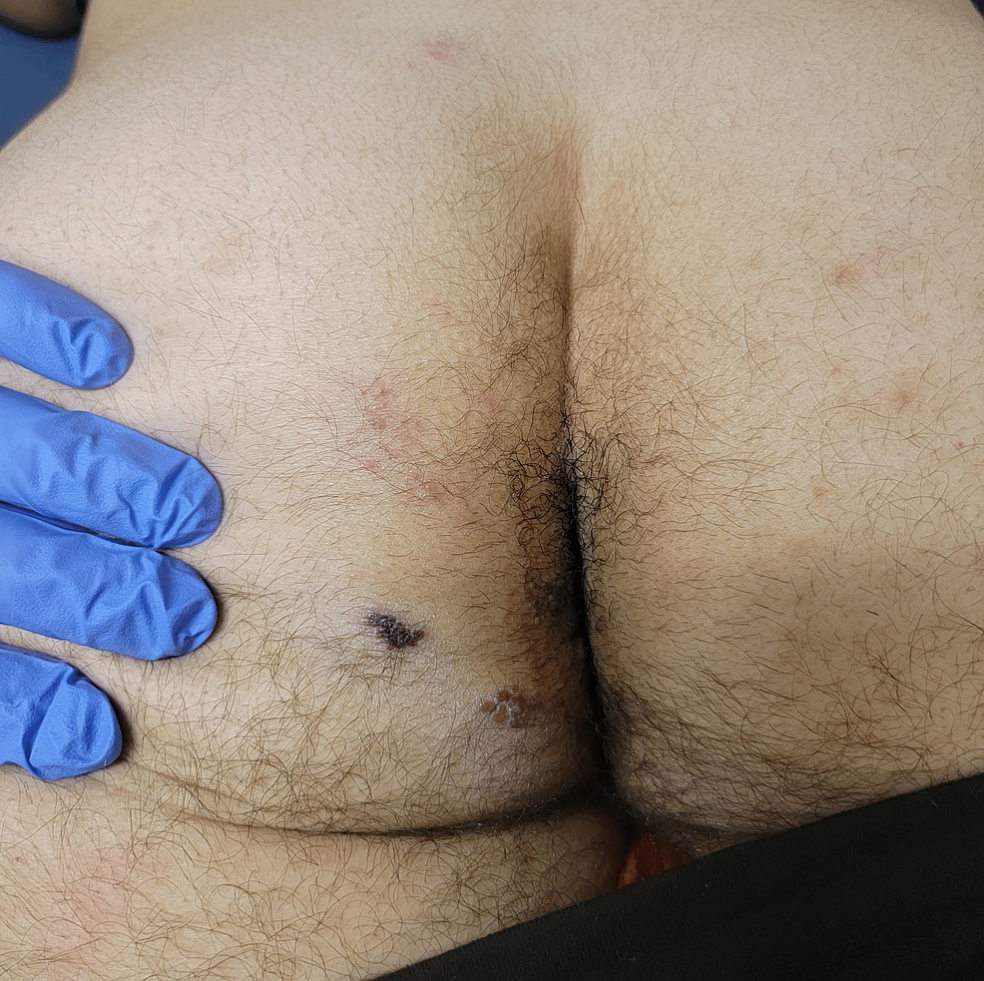
Maculopapular rash with coalescing lesions and scabbing in the S3-S4 dermatome.

Upon presentation, vitals showed tachycardia with a heart rate of 118 and tachypnea with a respiratory rate of 22. He was afebrile and had stable blood pressure. Physical examination was significant for nuchal rigidity and a maculopapular rash with coalescing lesions and scabbing over the S3-S4 dermatomal distribution (Figure 1).

Brudzinski's and Kernig’s signs were negative. Laboratory workup including complete blood count and basic metabolic panel were unremarkable. Computed tomography (CT) of the head showed no acute intracranial abnormality. CT of the cervical spine showed no osseous abnormalities. A lumbar puncture was performed, and cerebrospinal fluid (CSF) analysis suggested a diagnosis of viral meningitis with an elevated leukocyte count, increased protein, and a normal glucose level (Table 1).

**Table 1:** The patient's cerebrospinal fluid analysis with an elevated white blood cell count and protein along with a normal glucose value are suggestive of viral meningitis CSF: cerebrospinal fluid, WBC: white blood cell

Laboratory Marker	Patient’s Finding	Reference Range
CSF WBC count	174	0 - <10 cu/mm
CSF protein	136	15 - 45 mg/dL
CSF glucose	48	40 - 70 mg/dL

CSF polymerase chain reaction (PCR) assay revealed the presence of VZV. He was initiated on intravenous (IV) acyclovir for a total of 10 days. His symptoms improved significantly and was discharged on four additional days of valacyclovir 1 gram three times daily and advised to follow up with his primary care physician.

## Discussion

Aseptic meningitis is a broad term used to describe any cause of inflammation of the brain meninges in which a CSF culture does not demonstrate the presence of bacteria. VZV has been identified as the cause in 8-13% of viral meningitis cases. Meningitis is considered a rare complication of VZV and is only seen in 0.5% of reported infections, of which most patients are immunocompromised [3]. The precise mechanism by which VZV spreads to the central nervous system is still debated. One proposed theory is that the virus spreads through afferent nerve fibers and can transport to the meninges. Another theory suggests hematological spread causing infection of the central nervous system vasculature and neurons [3].

The development of neurological complications in our young, immunocompetent patient requires an in-depth review of possible risk factors making him more prone. Two of the most established risk factors are age and immune suppression. The incidence rate of herpes zoster is six to eight per 1000 person-years at the age of 60; at the age of 80, the rate increases to eight to 12 per 1000 person-years. Immune suppression in the form of human immunodeficiency virus (HIV), malignancy, chemotherapy, high-dose steroids, and biologics also increases the incidence [4]. A systematic review and meta-analysis were conducted in 2017 to further understand other risk factors for the development of herpes zoster. In the US and the UK, it was found that Caucasian and Asian individuals had a higher incidence rate of herpes zoster than black and Hispanic individuals. A history of herpes zoster in a first-degree blood relative has also been deemed a risk factor. Other risk factors identified include the female sex, autoimmune diseases, comorbidities such as chronic obstructive pulmonary disease (COPD), chronic kidney disease (CKD) and diabetes mellitus, physical trauma, and statin use [4]. Our patient had two of the above-mentioned risk factors being a Caucasian individual and having had a sister with herpes zoster in childhood. The genetic susceptibility of herpes zoster has been widely studied, specifically in families with no history of immunosuppression. Certain genetic polymorphisms affecting the functions of interleukin-10 have been described to predispose individuals to herpes zoster [5]. Our patient had no known personal or family history of immune deficiencies and autoimmune disorders, yet two members of the family developed a VZV infection at a young age which could signify a genetic predisposition to it.

The general recommendation for the treatment of VZV infection in immunocompetent patients is an initiation of antivirals within 72 hours of symptom onset or if new lesions continue to emerge [6]. Studies have demonstrated that oral valacyclovir has a higher bioavailability, better CSF penetration, and requires less frequent doses in comparison to oral acyclovir, thus it has become the oral antiviral of choice [7]. Our patient was initiated on oral valacyclovir on day three of symptoms according to the standard herpes zoster dosage despite which he went on to develop meningitis two days later. Another case report also outlines a patient who developed meningitis following herpes zoster two days after being started on a standard dosage of oral valacyclovir [6]. Other case reports have demonstrated successful management of VZV meningitis with oral valacyclovir however, in both cases the patient first received intravenous acyclovir followed by a higher than standard dosage of oral valacyclovir [2,8].

## Conclusions

VZV reactivation has the potential to lead to several neurological complications, despite the use of oral antiviral therapy. The ideal treatment to reduce hospital stay, optimize the cost of care and ensure adequate results is the initiation of intravenous acyclovir followed by oral valacyclovir at the higher dosage. With our case, we would also like to emphasize that physicians should be aware of risk factors for the development of VZV infection beyond the widely known conditions of older age and immune suppression. Our patient with a first-degree relative with a history of herpes zoster infection was at a higher risk of developing the same. If his family history had been explored earlier on in his disease course, his neurological complication could have been better anticipated and possibly prevented.
